# Therapeutic efficacy of anti‐MMP9 antibody in combination with nab‐paclitaxel‐based chemotherapy in pre‐clinical models of pancreatic cancer

**DOI:** 10.1111/jcmm.14242

**Published:** 2019-04-02

**Authors:** Niranjan Awasthi, Amanda J. Mikels‐Vigdal, Erin Stefanutti, Margaret A. Schwarz, Sheena Monahan, Victoria Smith, Roderich E. Schwarz

**Affiliations:** ^1^ Department of Surgery Indiana University School of Medicine South Bend Indiana; ^2^ Harper Cancer Research Institute University of Notre Dame South Bend Indiana; ^3^ Gilead Sciences Inc. Foster City California; ^4^ Department of Pediatrics Indiana University School of Medicine South Bend Indiana

**Keywords:** combination therapy, gemcitabine, MMP9, *nab*‐paclitaxel, pancreatic cancer

## Abstract

Matrix metalloproteinase 9 (MMP9) is involved in the proteolysis of extracellular proteins and plays a critical role in pancreatic ductal adenocarcinoma (PDAC) progression, invasion and metastasis. The therapeutic potential of an anti‐MMP9 antibody (αMMP9) was evaluated in combination with nab‐paclitaxel (NPT)‐based standard cytotoxic therapy in pre‐clinical models of PDAC. Tumour progression and survival studies were performed in NOD/SCID mice. The mechanistic evaluation involved RNA‐Seq, Luminex, IHC and Immunoblot analyses of tumour samples. Median animal survival compared to controls was significantly increased after 2‐week therapy with NPT (59%), Gem (29%) and NPT+Gem (76%). Addition of αMMP9 antibody exhibited further extension in survival: NPT+αMMP9 (76%), Gem+αMMP9 (47%) and NPT+Gem+αMMP9 (94%). Six‐week maintenance therapy revealed that median animal survival was significantly increased after NPT+Gem (186%) and further improved by the addition of αMMP9 antibody (218%). Qualitative assessment of mice exhibited that αMMP9 therapy led to a reduction in jaundice, bloody ascites and metastatic burden. Anti‐MMP9 antibody increased the levels of tumour‐associated IL‐28 (1.5‐fold) and decreased stromal markers (collagen I, αSMA) and the EMT marker vimentin. Subcutaneous tumours revealed low but detectable levels of MMP9 in all therapy groups but no difference in MMP9 expression. Anti‐MMP9 antibody monotherapy resulted in more gene expression changes in the mouse stroma compared to the human tumour compartment. These findings suggest that anti‐MMP9 antibody can exert specific stroma‐directed effects that could be exploited in combination with currently used cytotoxics to improve clinical PDAC therapy.

## 1 INTRODUCTION

Pancreatic ductal adenocarcinoma (PDAC) is one of the most aggressive tumours and is characterized by extensive local invasion, metastasis to distant organs and high rate of treatment failure after both local and systemic therapies.^1^ Through its extremely poor prognosis, PDAC is estimated to become the second leading cause of cancer‐associated mortality by 2030.^2^ This dismal outcome in PDAC is in part related to the absence of early diagnostic markers, aggressive progression pattern and lack of effective therapeutic options. While surgical resection remains the only curative treatment option for PDAC, unfortunately, only 15%‐20% of patients are candidates for resection, and most resected PDAC patients succumb to disease recurrence.^3^ Therefore, in recent years, much attention has been placed on improving systemic therapy options for PDAC. Gemcitabine (Gem), a nucleoside pyrimidine analogue, became the standard drug in PDAC after a positive clinical trial in 1997; it demonstrated a 5%‐10% response rate and a median overall survival of 6 months.^4^ FOLFIRINOX treatment, a combination of three chemotherapy drugs, nearly doubled the overall patient survival, but this regimen has higher toxicity risks.^5^ Nab‐paclitaxel (NPT) in combination with gemcitabine (Gem) is currently the most widely used chemotherapy regimen due to its favourable toxicity profile and median overall survival 8.5 months.^6^ Due to these limitations of current regimens for clinical PDAC therapy, there is an urgent requirement for novel therapeutic strategies with better efficacy and less toxicity.

Matrix metalloproteinases (MMPs) are a family of zinc‐containing enzymes that degrade extracellular matrix (ECM) components and play a crucial role in tumour invasion, metastasis and angiogenesis.^7^ MMPs comprise a large family of 23 members that can be subclassified into different groups mainly based on their substrate specificity and amino acid sequence including collagenases (MMP‐1, 8, 13), gelatinases (MMP‐2, 9), stromelysins (MMP‐3, 10, 11), matrilysin (MMP‐7), metalloelastase (MMP‐12) and membrane‐bound proteinases (MMP‐14, 15, 16).^8^ The activity of MMPs is tightly regulated by their endogenous inhibitors, the tissue inhibitors of metalloproteinase (TIMPs). MMP expression is up‐regulated in several solid tumours including pancreatic cancer and correlates with tumour invasiveness and metastatic potential.^9^, ^10^ Several small molecule semi‐selective MMP inhibitors have been studied in different solid tumours including pancreatic cancer. A broad‐spectrum MMP inhibitor BB‐94 demonstrated a significant antitumour response in pre‐clinical pancreatic cancer models.^11^ However, in clinical studies, the broad‐spectrum MMP inhibitors marimastat or tanomastat failed to show any significant clinical response.^12^, ^13^ The failure of the broad‐spectrum MMP9 inhibitors in clinical trials was mainly correlated with dose‐limiting side effects, a narrow therapeutic window as MMPs play a critical role in homeostatic processes 14 and general lack of efficacy in advanced tumour burden settings.^15^, ^16^ Therefore, recently, the focus has been shifted towards more specific MMP inhibitors (such as MMP2 or 9 antibodies) that may have better efficacy and improved toxicity profile.

Matrix metalloproteinase 9 (MMP9) is one of the type IV collagenases of the MMP family that is capable of cleaving a wide range of ECM components including gelatins, denatured collagens, elastin and laminin.^17^ MMP9 is involved in many developmental processes, including ECM degradation, angiogenesis and wound healing. Recently, MMP9 has also been shown to be involved in cell proliferation, migration, invasion and epithelial‐mesenchymal transition (EMT).^18^ MMP9 overexpression has been observed in many solid tumours, including pancreatic cancer, and is correlated with poor prognosis.^19^, ^20^, ^21^, ^22^, ^23^, ^24^, ^25^ In the present study, we evaluated the therapeutic efficacy of a highly selective and potent anti‐MMP9 antibody AB0046, the murine surrogate of humanized MMP9 antibody andecaliximab, in combination with nab‐paclitaxel‐based standard chemotherapy in pre‐clinical models of pancreatic cancer. Previous studies have shown the antitumour efficacy of AB0046 in pre‐clinical models of colorectal cancer 26 and encouraging clinical activity of andecaliximab in combination with chemotherapy in gastric cancer.^27^

## 2 MATERIALS AND METHODS

### 2.1 Cell culture and reagents

The human PDAC cell line AsPC‐1 was purchased from the American Type Culture Collection (ATCC, Rockville, MD) and had been tested and authenticated by ATCC. Characteristic genetic alterations in the AsPC‐1 cell line include KRAS activating mutation, p53 inactivating mutation and p16 homozygous deletion. The cell line was used within 6 months after reexpansion in culture. Cells were cultured in RPMI 1640 medium (Sigma Chemical Co., St. Louis, MO) containing 10% FBS and maintained at 37°C in a humidified incubator with 5% CO_2_ and 95% air. Nab‐paclitaxel was obtained from Celgene Corporations (Summit, NJ). Mouse monoclonal anti‐MMP9 antibody AB0046 26 was obtained from Gilead Sciences (Foster City, CA).

### 2.2 Immunoblot analysis

Tumours obtained from intraperitoneal or subcutaneous xenografts were sectioned, snap frozen in liquid nitrogen and stored at −80°C. Protein lysates of these tumour sections were prepared by suspending in lysis buffer and homogenizing using the Bullet Blender Homogenizer (Next Generation, Averill Park, NY), and extracts were sonicated on ice. Proteins in supernatants were separated by SDS‐PAGE and transferred to polyvinylidene difluoride membranes (Bio‐Rad, Hercules, CA). The membranes were incubated overnight at 4°C with antibodies against vimentin, α‐smooth muscle actin (α‐SMA), phospho‐stathmin, VEGF, IL‐6 and GAPDH (Cell Signaling Technology, Beverly, MA). The membranes were then incubated with the corresponding horseradish peroxidase‐conjugated secondary antibodies (Pierce Biotechnologies, Santa Cruz, CA) for 1 hour. Protein bands were visualized with an Image360 system after using the enhanced chemiluminescence reagent (SignalFire, Cell Signaling) and quantitated by densitometry.

### 2.3 Immunohistochemistry and immunofluorescence

Standard immunohistochemistry protocol was followed to stain the tumour tissue sections, as previously described.^28^ Briefly, tumour tissues fixed in 4% paraformaldehyde were dehydrated in a graded series of ethanol and deparaffinized with xylene. The tumour tissues were then embedded in paraffin and cut into 5 μm sections using microtome. The tumour tissue sections were then deparaffinized and rehydrated through graded ethanol followed by heat‐mediated antigen retrieval using citrate buffer. The tissue sections were incubated for 20 minutes in CAS blocking buffer followed by overnight incubation at 4°C with 1:200 dilution of primary antibodies against endomucin (MAB2624, Millipore), collagen I (ab6308, Abcam, Cambridge, MA), vimentin (#5741, Cell Signaling) or α‐SMA (C6198, Sigma). The tissue sections were washed with PBS and incubated with 1:200 dilution of secondary antibody conjugated with Cy3 (Jackson ImmunoResearch Laboratories, West Grove, PA) or Alexa Fluor 488 (Life Technologies, Grand Island, NY) at room temperature for 40 minutes to visualize the antigen. Tissues were then washed and mounted with a mounting solution containing 4′,6‐diamidino‐2‐phenylindole (DAPI) (Invitrogen, Carlsbad, CA) to visualize nuclei. Fluorescence microscopy was used to detect fluorescent signals in five representative high‐power field (HPF) per sample using IX81 Olympus microscope and images were captured with a Hamamatsu Orca digital camera (Hamamatsu Corporation, Bridgewater, NJ) with a DSU spinning confocal unit using cellSens Dimension software (Olympus, Center Valley, PA).

### 2.4 Luminex assay

Protein analysis of tumour samples from the intraperitoneal xenografts was performed by Luminex assay. Briefly, lysates were prepared using the OMNI bead ruptor homogenizer (OMNI International, Kennesaw, GA) in RIPA buffer containing benzonase and protease/phosphatase inhibitors. Samples were then centrifuged at 14 000 *g* for 10 minutes. The supernatant was transferred to a new tube and total protein content was measured. Luminex analysis of these samples was performed with rodent MAP 4.0 mouse panel at Ampersand Biosciences (Saranac Lake, NY).

### 2.5 RNA‐Seq analysis

Gene expression changes in different therapy groups were determined by RNA sequencing (RNA‐Seq) of tumour samples from subcutaneous xenografts. RNA samples isolated from frozen tumours using a Qiagen RNAeasy kit (Qiagen, Germantown, MD) were converted into cDNA libraries using the Illumina TruSeq Stranded mRNA sample preparation kit (Illumina #RS‐122‐2103, San Diego, CA), and RNA‐Seq was performed (Q2 Solutions, Morrisville, NC). The raw fastq files were first run through FastQC to verify the data were of high quality and processed using the Expression analysis mRNAv9‐RSEM pipeline. After removing sequencing adapters and other low‐quality bases, the clipped fastq files were aligned to the mouse reference genome (build GRCm38) using STAR v2.4. The resulting BAM files were fed into the quantification software, RSEM v1.2.14. RSEM output the counts of the sequencing reads for each gene and sample. After normalization, we performed quality control analyses of QC'd, the data set to identify strong batch effects and outlier samples using principal component analysis and sample dendrogram. Genes with <1 sequencing read count/106 (CPM) in ≥3 samples were removed as low count genes. Generalized linear regression in edgeR was used to estimate log2 fold changes and *P* values. The *P* values were adjusted using the false discovery rate control by following the Benjamini‐Hochberg procedure. Next, the estimated *P* values of all the genes were converted to z scores using the zScores function in the R package gCMAP. The z scores were used to rank the list of genes, which was analysed with GSEAPreranked included in the Broad GSEA Java tool for Gene set enrichment analysis against MSigDB, C2 (curated gene sets), C6 (oncogenic signatures) and C7 (immunologic signatures) collections.

### 2.6 Subcutaneous xenograft studies

All animals were housed in a pathogen‐free facility with access to food and water ad libitum. Animal experiments were performed in accordance with the Institutional Animal Care and Use Committee (IACUC) at the Indiana University School of Medicine (South Bend, IN). Female non‐obese diabetic/severe combined immunodeficient (NOD/SCID) mice (4‐6 weeks old) were subcutaneously injected with AsPC‐1 cells (7.5 × 10^5^) as previously described.^29^ Two weeks after tumour cell injection, all mice had a measurable tumour. Mice were then randomized (n = 5 per group) to receive PBS (control), nab‐paclitaxel (5 mg/kg, twice a week), gemcitabine (50 mg/kg, twice a week) and anti‐MMP9 antibody (50 mg/kg bolus dose on day 1, then 20 mg/kg twice a week) via intraperitoneal injection for the next 2 weeks. The tumour size was measured twice weekly, and tumour volume (V) was calculated using the formula V  =  ½ (Length x Width^2^). Mice were killed after completion of treatment, tumours were dissected, weighed and processed for histological, immunoblot and RNA‐Seq analysis.

### 2.7 Peritoneal dissemination animal studies

Animal survival studies were performed with female NOD/SCID mice (4‐6 weeks of age) as previously described.^30^ Briefly, the mice were injected intraperitoneally with AsPC‐1 cells (0.75 × 10^6^ or 0.65 × 10^6^) and 2 weeks after tumour cell injection, mice were randomized (n = 5‐7 per group) to receive PBS (control), nab‐paclitaxel (5 mg/kg, twice a week), gemcitabine (50 mg/kg, twice a week) and anti‐MMP9 antibody (50 mg/kg bolus dose on day 1, then 20 mg/kg twice a week) via IP injection for the next 2 or 6 weeks. Animals were killed when moribund according to predefined criteria.^31^, ^32^ Animal survival was evaluated from the first day of treatment until death. For qualitative assessment of mice in the peritoneal dissemination xenograft study, five mice in each therapy group were evaluated for jaundice, ascites, tumour burden and metastasis when control mice became moribund. The tumours obtained from these mice were processed for Luminex, IHC and Immunoblot analysis.

### 2.8 Statistical analysis

Statistical analysis for in vivo tumour growth studies was performed by one‐way ANOVA for multiple group comparisons and Student's *t* test for the individual group comparisons. Survival study statistics were performed with logrank group comparison (GraphPad Prism 6.0, San Diego, CA). We used G*Power 3.1 software for the power calculation. With sample size of 5‐7 mice per group, a preset α value 0.05 and anticipated change in animal survival or tumour size of 40% or more and a standard deviation of 20%, the expected power was more than 80% to detect statistically significant differences. Luminex assay data were analysed using unpaired *t* test with Welch's correction or Mann‐Whitney rank sum tests (GraphPad Prism). *P* < 0.05 was considered statistically significant.

## 3 RESULTS

### 3.1 Improvement in animal survival

**Figure 1:**
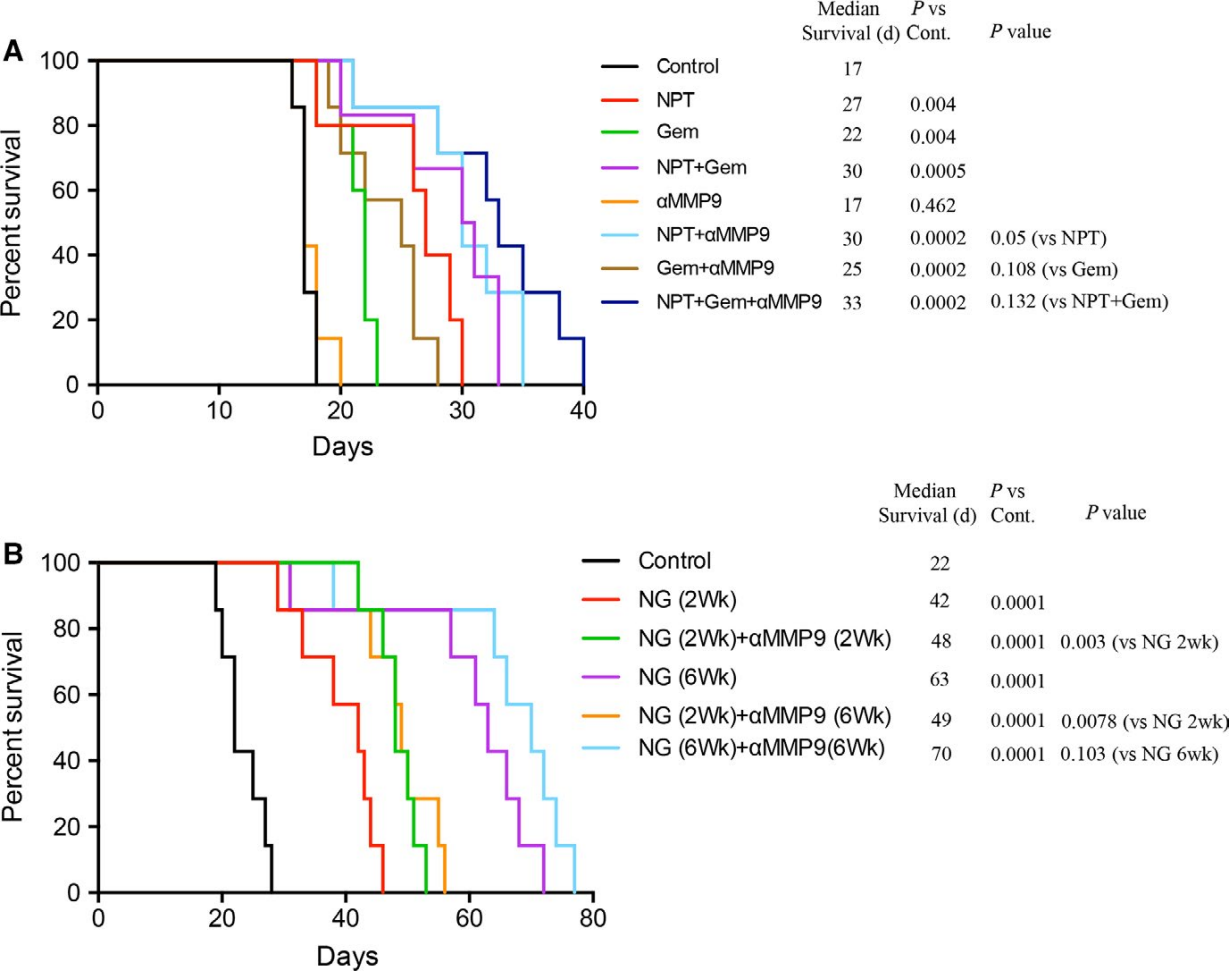
Anti‐MMP9 antibody improves animal survival benefits of nab‐paclitaxel/gemcitabine. Animal survival study in the peritoneal dissemination model after injecting 0.75 × 10^6^ AsPC‐1 cells (A) or 0.65 × 10^6^ AsPC‐1 cells (B). Two weeks after tumour cell injection in NOD/SCID mice, treatment was started with *nab*‐paclitaxel, gemcitabine and anti‐MMP9 antibody for 2 wk or 6 wk as specified. The curve represents the animal survival time from the beginning of therapy. Statistical group differences in survival time were calculated using logrank testing

In an AsPC‐1 PDAC peritoneal dissemination xenograft study with 14‐day therapy course (injected AsPC‐1 cells 0.75 × 10^6^), median animal survival compared to controls (17 days) was increased after therapy with NPT (27 days, a 59% increase), Gem (22 days, a 29% increase) and NPT+Gem (30 days, a 76% increase). Addition of anti‐MMP9 antibody increased survival as follows: NPT+αMMP9 (30 days, a 76% increase), Gem+αMMP9 (25 days, a 47% increase) and NPT+Gem+αMMP9 (33 days, a 94% increase) (Figure 1A). Another AsPC‐1 PDAC peritoneal dissemination xenograft study (injected AsPC‐1 cells 0.65 × 10^6^) to evaluate the efficacy of maintenance therapy revealed that median animal survival (controls: 22 days) was increased after 2‐week therapy with NPT+Gem (42 days, a 91% increase). Combination of 2‐week anti‐MMP9 therapy increased this survival to 48 days (a 118% increase). Six‐week maintenance therapy with NPT+Gem led to a median survival of 63 days (a 186% increase) that was further improved by the addition of 6‐week anti‐MMP9 antibody therapy to 70 days (a 218% increase). However, a combination of 6‐week maintenance therapy of anti‐MMP9 antibody with 2 weeks of NPT+Gem (49 days) could not establish any survival benefit over 2‐week NPT+Gem+αMMP9 therapy (48 days) (Figure 1B). No significant change in the bodyweight of mice was observed during 2 or 6‐week therapy period, indicating that there was no discernable therapy‐associated toxicity in all therapy groups.

### 3.2 Qualitative assessment of tumour‐associated aspects

**Figure 2:**
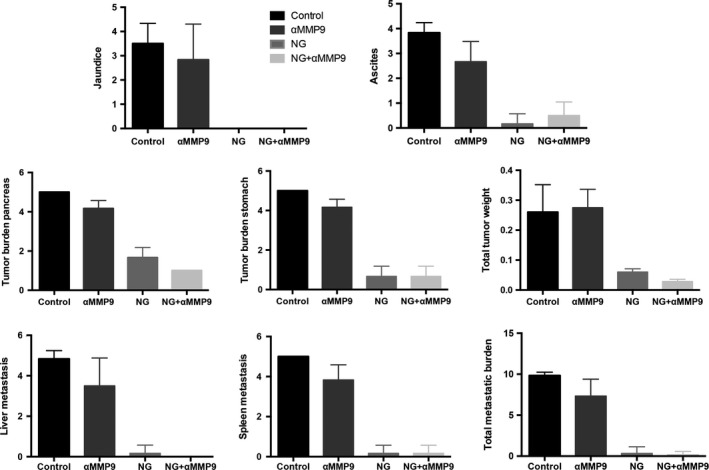
Qualitative assessment of tumour‐associated aspects after therapy with anti‐MMP9 antibody and *nab*‐paclitaxel‐based chemotherapy. In an AsPC‐1 PDAC peritoneal dissemination xenograft study with 2‐wk therapy schedule, qualitative assessment of mice in all therapy groups was performed when control mice became moribund including jaundice, amount of ascites, tumour burden in the pancreas, tumour burden in the stomach, total tumour weight, liver metastasis, spleen metastasis and total metastatic burden

In an AsPC‐1 PDAC peritoneal dissemination xenograft study with 2‐week therapy schedule, qualitative assessment of mice in all therapy groups was performed when control mice became moribund. Evaluation of different tumour‐associated findings revealed that compared with controls, NPT+Gem therapy led to a significant reduction in all aspects observed including jaundice, amount of ascites, tumour burden in the pancreas, tumour burden in the stomach, total tumour weight, liver metastasis, spleen metastasis and total metastatic burden. Anti‐MMP9 antibody therapy significantly decreased ascites, tumour burden in pancreas and stomach, liver metastasis, spleen metastasis and total metastatic burden, as compared with controls. However, most of the findings after NPT+Gem+αMMP9 therapy were not significantly different from those after NPT+Gem therapy alone, except a decrease in tumour burden in the pancreas (Figure 2).

### 3.3 Changes in protein expression in tumours from intraperitoneal xenografts

**Figure 3:**
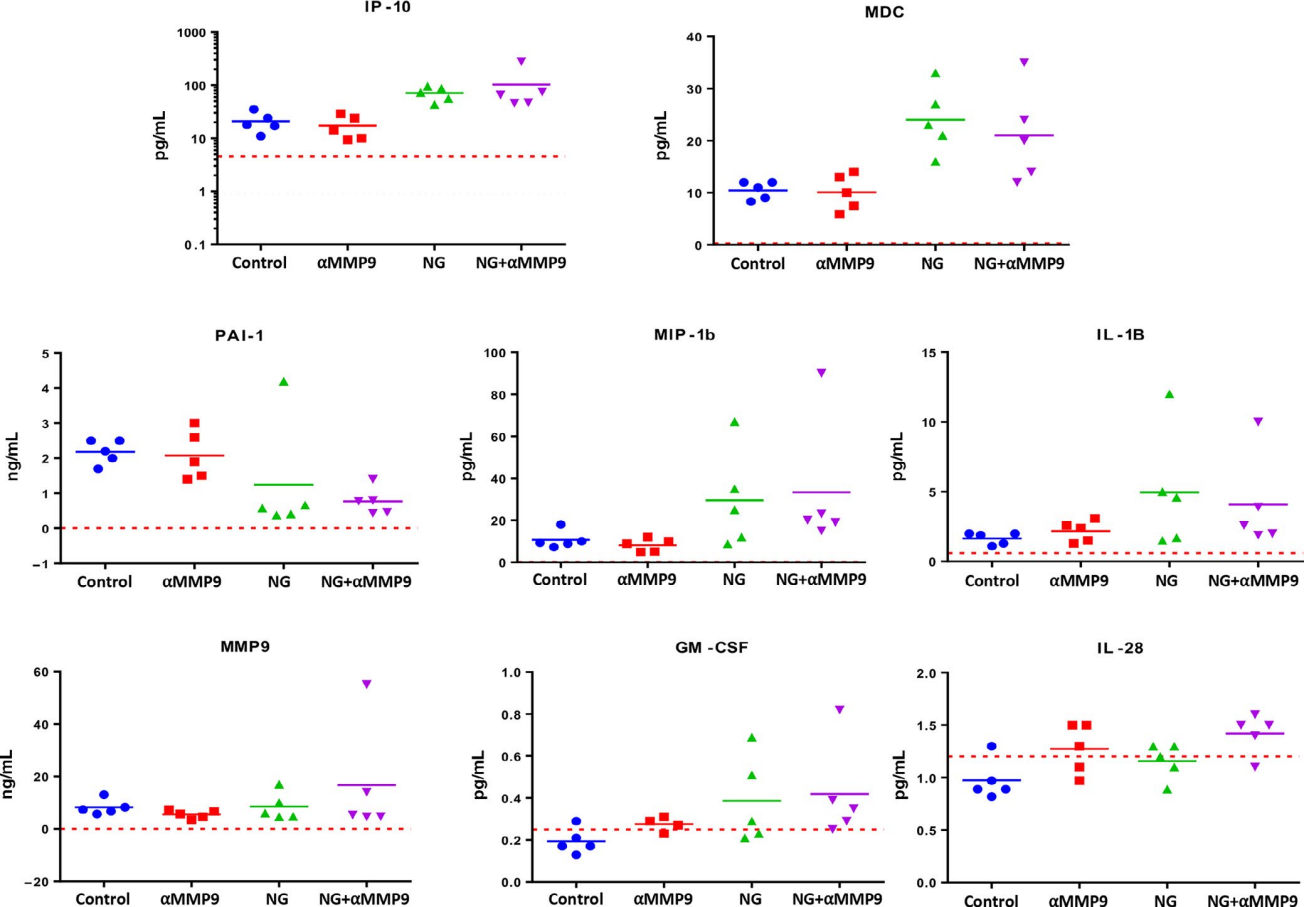
Luminex analysis of cytokine and growth factor expression after treatment with anti‐MMP9 antibody and *nab*‐paclitaxel‐based chemotherapy. The Luminex assay was performed in tumour lysates from AsPC‐1 PDAC intraperitoneal xenografts. The red lines denote the lowest limit of detection for the analysis. The data were analysed using unpaired t test with Welch's correction or Mann‐Whitney rank sum tests (GraphPad Prism)

Luminex analysis of cytokine or growth factor expression in tumour lysates from AsPC‐1 PDAC peritoneal dissemination xenografts demonstrated NPT+Gem therapy‐induced changes in most studied marker proteins including IP‐10, MDC, PAI‐1, MIP‐1b, IL‐1B and GM‐CSF. An increase in IL28 expression (1.5‐fold, *P* = 0.016) was specifically related to anti‐MMP9 antibody therapy as it was significantly higher after both αMMP9 monotherapy and NPT+Gem+αMMP9 combination compared to controls or NPT+Gem alone (Figure 3).

**Figure 4:**
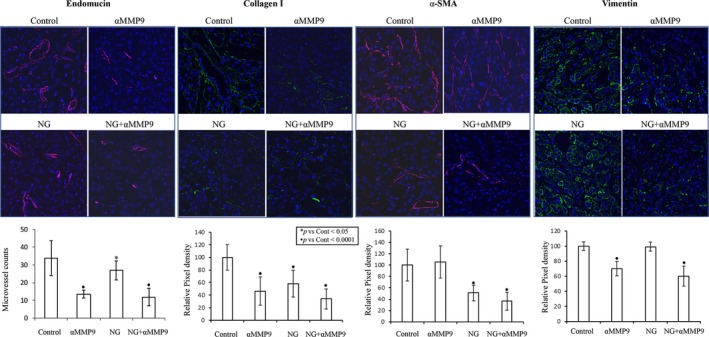
IHC analysis of stromal and EMT markers after treatment with anti‐MMP9 antibody and *nab*‐paclitaxel‐based chemotherapy. Tumour tissue sections obtained from the AsPC‐1 PDAC peritoneal xenograft study were analysed by IHC. Tissue sections were immunostained with antibodies to determine tumour vasculature (endomucin), stromal markers (collagen‐I, SMA) and EMT marker (vimentin) and slides were photographed under a fluorescent microscope. Positive staining was calculated within a microscopic HPF in a blinded manner and the data are expressed as the mean ± SD

**Figure 5:**
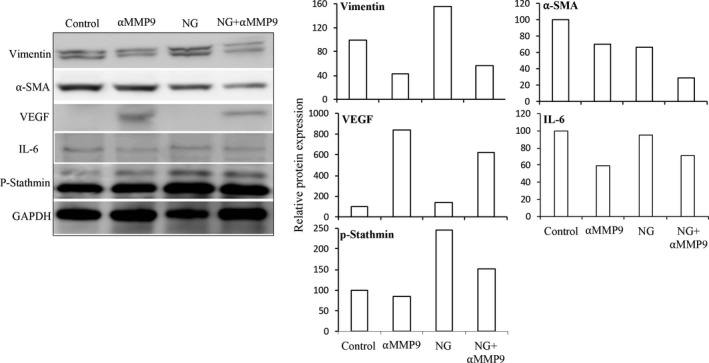
Immunoblot analysis of stromal and EMT markers after treatment with anti‐MMP9 antibody and *nab*‐paclitaxel‐based chemotherapy. Protein lysates of tumours obtained from intraperitoneal xenografts were separated by SDS‐PAGE and the membranes were incubated with antibodies against vimentin, α‐SMA, VEGF, IL‐6, phospho‐stathmin and GAPDH. A, Protein bands were visualized with an Image360 system after using the enhanced chemiluminescence reagent. B, The intensity of bands was quantitated by densitometry and is represented in the bar graph after normalizing values with GAPDH expression

Anti‐MMP9 antibody related changes in the expression of tumourigenic markers were further determined in tumour sections from AsPC‐1 PDAC peritoneal xenografts by IHC analysis. Endomucin, a vascular endothelial marker, staining for determining tumour microvessel density demonstrated a 21% decrease with NPT+Gem, 60% decrease with αMMP9 and 65% decrease with NPT+Gem+αMMP9 therapy (Figure 4). Further, αMMP9 and NPT+Gem+αMMP9 therapy reduced the expression of stromal marker protein collagen I by 53% and 66%; and EMT marker protein vimentin by 30% and 40% respectively. The other stromal marker protein αSMA was significantly decreased by NPT+Gem and NPT+Gem+αMMP9 therapy groups by 49% and 64% respectively (Figure 4). Furthermore, Immunoblot analysis of lysates from AsPC‐1 PDAC peritoneal dissemination xenograft study showed decreased vimentin expression (>42.5%) and IL‐6 expression (>29%) in αMMP9 therapy groups. The expression of αSMA was significantly reduced by NPT+Gem chemotherapy while it was only slightly decreased by αMMP9. VEGF expression was increased by αMMP9 antibody treatment but not by NPT/Gem therapy (Figure 5).

### 3.4 Gene expression changes in the tumour microenvironment in subcutaneous xenografts

**Figure 6:**
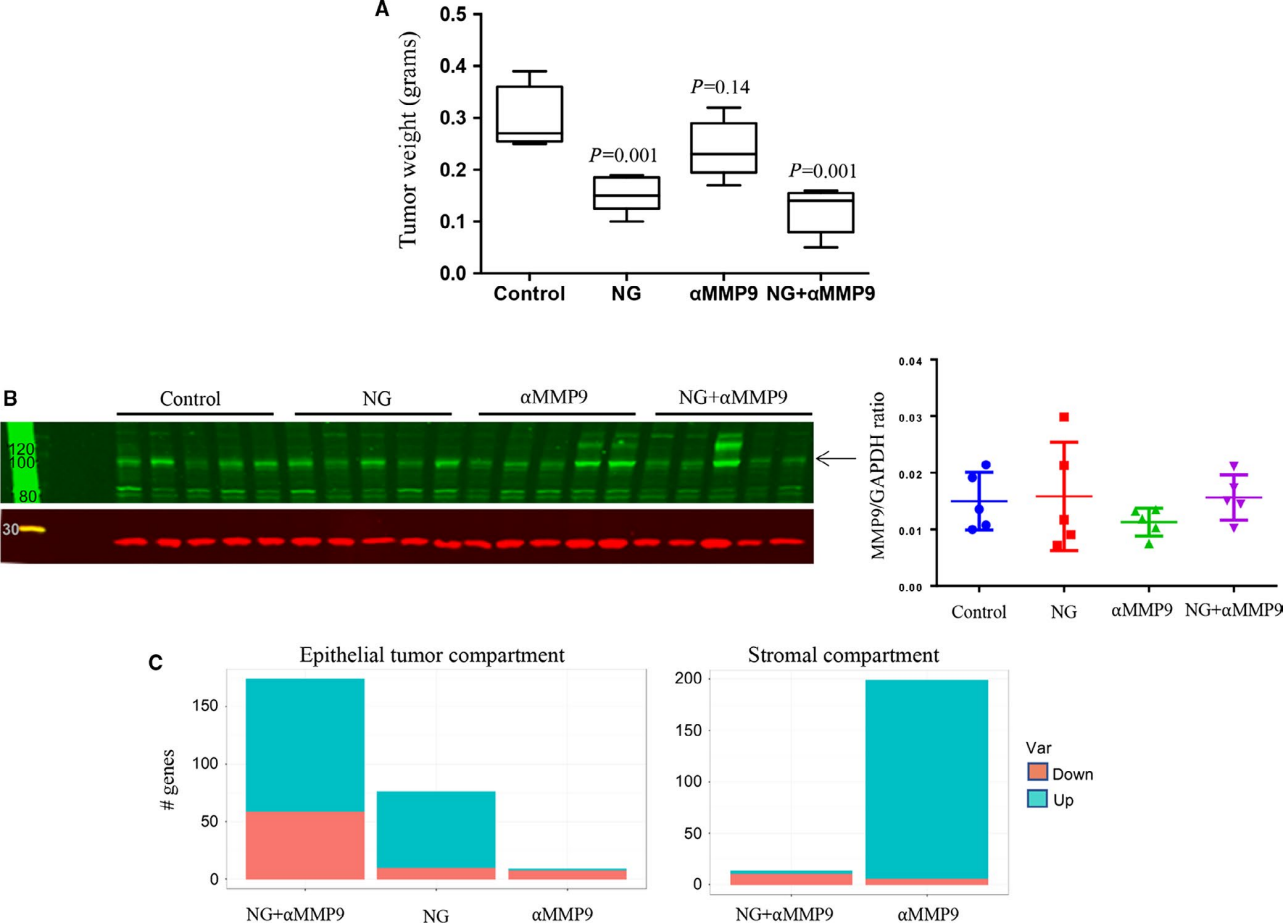
Effect of anti‐MMP9 antibody and nab‐paclitaxel‐based chemotherapy in AsPC‐1 subcutaneous xenografts. A, Mean tumour weight was calculated from final day tumour weights in each group and presented as a Box plot (B), Quantitative Western blot analysis of tumour tissues for MMP9 protein expression using anti‐MMP9 antibody and secondary antibody conjugated with fluorescent dye (C), RNA‐Seq analysis of tumour tissues for gene expression changes in different compartments of the tumour microenvironment

In an AsPC‐1 subcutaneous xenograft study, compared with controls, mean tumour weight after 2‐week therapy decreased by 50% with NPT+Gem (*P* = 0.001), 20% with αMMP9 (*P* = 0.136) and 63% with NPT+Gem+αMMP9 (*P* = 0.0006) (Figure 6A). The difference in tumour weight between NPT+Gem and NPT+Gem+αMMP9 did not reach statistical significance (Figure 6A). Tumour tissues from these subcutaneous xenografts revealed low but detectable levels of MMP9 protein in all therapy groups without any significant difference in MMP9 expression (Figure 6B). In addition, RNA‐Seq analysis to determine numeric gene expression changes in different compartments of the tumour microenvironment showed that αMMP9 monotherapy, using AB0046 which only inhibits murine MMP9, resulted in more gene expression changes in the mouse stroma than in the human epithelial tumour compartment, compared to other treatments. In contrast, NPT+Gem+αMMP9 combination therapy resulted in greater numbers of changes in gene expression compared to the other treatments in the tumour compartment (Figure 6C).

As in prior experiments, no discernable therapy‐related toxicity was observed in the subcutaneous xenograft study during the therapy period as there was no significant change in the mouse bodyweight in all groups (Figure S1).

## 4 DISCUSSION

The hallmarks of pancreatic cancer are an aggressive local growth of tumour into surrounding parenchyma, vascular and other peripancreatic structures, accompanied by rapid metastatic tumour progression. Many of these phenomena require degradation of the surrounding ECM proteins by MMPs. Recent studies have shown an essential role of MMP9 in tumour progression of many solid tumours including PDAC through modulating stromal tumour microenvironment promoting angiogenesis, metastasis, EMT and drug resistance.^9^, ^10^, ^18^, ^25^, ^33^ Given the critical role of tumour stroma in PDAC progression, metastasis and therapy resistance, it is sensible to explore combination approaches of promising cytotoxic and antistromal components to accomplish superior therapy benefits. The present study was undertaken to determine the target engagement of a novel, highly specific anti‐MMP9 antibody within the PDAC tumour microenvironment, and to define its therapeutic efficacy in combination with nab‐paclitaxel‐based cytotoxic chemotherapy in pre‐clinical PDAC models.

Peritoneal dissemination is a frequent incidence in PDAC, which is associated with a poor prognosis.^34^ The peritoneal dissemination animal survival model used in this study can serve as a relevant pre‐clinical model of PDAC as it involves the formation of desmoplastic stroma and metastasis to liver and other visceral sites.^35^ Although the present study is based on animal experiments using one human PDAC cell line AsPC‐1, it is well‐established and a good representative of clinical PDAC in terms of oncogenic mutations (KRAS, p53 and p16), aggressiveness and chemoresistance. In the present study, a modest but reproducible and at times significant effect of anti‐MMP9 antibody to improve survival benefits of NPT‐based chemotherapy regimens correlates well with its down‐regulatory effects on stromal and EMT markers such as endomucin, collagen I and vimentin. Furthermore, increased levels of IL28 are quite likely a specific effect of anti‐MMP9 therapy that might also be partaking in extending survival benefits of NPT‐based chemotherapy, because a previous study demonstrated an antitumour impact of IL28 against human lung cancer cells.^36^ Anti‐MMP9 antibody therapy survival benefits correlated well with the qualitative assessment of mice in terms of jaundice, ascites, pancreatic tumour mass and metastatic burden. Decreased tumour angiogenesis as observed by reduced tumour microvessel density in peritoneal dissemination xenografts after anti‐MMP9 antibody therapy may be unrelated to the increase in VEGF expression. This likely reflects a stromal response that stands in contrast to some published reports indicating a decrease in VEGF by MMP9 inhibition.^37^ In this model system, we suspect that the VEGF increase is a compensatory mechanism due to the specific antiangiogenic effect of anti‐MMP9 therapy.

Subcutaneous xenograft studies to verify target (MMP9) expression and evaluate therapy effects in different compartments of the tumour microenvironment demonstrated maximum gene expression changes within the tumour stroma by anti‐MMP9 antibody additionally supporting its antistromal activity in the PDAC tumour microenvironment. However, in contrast to the peritoneal dissemination tumour model, the efficacy of anti‐MMP9 antibody in the subcutaneous was reduced. This can likely be attributed to the absence of tumour progression steps leading to metastasis in subcutaneous xenografts, and to possible differences in the desmoplastic reaction between the two models that affect the efficacy of anti‐MMP9 stromal targeting. In this context, we do consider the peritoneal dissemination tumour model more clinically relevant to PDAC compression compared with the subcutaneous tumour model.

Due to the multifactorial effects of nab‐paclitaxel‐based chemotherapy and anti‐MMP9 antibody on PDAC progression, it is likely that specific molecular mechanisms of these two regimens regulate the potential of combination therapy. We have previously observed a correlation between antitumour benefits of nab‐paclitaxel‐based chemotherapy and its antimitotic and antistromal effects in PDAC.^29^, ^38^ The advantages of nab‐paclitaxel‐based chemotherapy can likely be attributed to better drug distribution, bioavailability, tumour penetration and higher retention of itself and other drugs used in combination. Although the exact mechanisms for the augmentation of nab‐paclitaxel‐based chemotherapy by anti‐MMP9 antibody remain indistinct, these can be correlated with normalization or reduction in tumour vasculature, depletion of tumour stroma enhancing bioavailability of chemotherapy drugs and direct inhibition of ECM degradation leading to antimetastatic effects.^26^, ^39^, ^40^

Multiple mechanisms are involved in PDAC growth and progression including an increase in tumour cell proliferation, differentiation, invasion, migration, angiogenesis and EMT. Therefore, a more effective therapeutic regimen would likely impact on most of these pro‐tumourigenic mechanisms with manageable toxicity. Based on the negative impact of anti‐MMP9 antibody in the pre‐clinical PDAC models in terms of tumour stroma, metastasis and EMT, our data support the possible advantages of anti‐MMP9 antibody therapy in PDAC patients with localized non‐metastatic disease. Importantly, clinical failure of early broad‐spectrum MMP inhibitors was correlated with a large number of patients with metastatic disease.^12^, ^13^, ^25^ Recently, anti‐MMP9 antibody has also been shown to promote antitumour immunity by activating T cells in the gastric cancer microenvironment.^41^ Overall, the present study demonstrates that with an anti‐MMP9 antibody, direct enhancement of PDAC cytotoxic therapy is not high but the specific mechanistic footprint of MMP9 inhibition should be explored for its contribution benefit to combinations with antivascular, other antistromal and immune‐based therapy.

## ACKNOWLEDGEMENTS

This work was financially supported by Gilead Sciences grant to N. Awasthi and R.E. Schwarz, Indiana University School of Medicine funds to R.E. Schwarz. We thank Carrie Brachman and Juliane Jurgensmeier of Gilead Sciences for the critical review of this manuscript.

## CONFLICT OF INTERESTS

The authors N. Awasthi and R.E. Schwarz received funding support from Gilead Sciences to perform this research. The authors A. J. Mikels‐Vigdal, E. Stefanutti and V. Smith are employees of Gilead Sciences. The authors M.A. Schwarz and S. Monahan declare no conflict of interest.

## Supporting information


** **

** **

** **
