# Supplementary figures and images for: Therapeutic efficacy of anti‐MMP9 antibody in combination with nab‐paclitaxel‐based chemotherapy in pre‐clinical models of pancreatic cancer
Source: J Cell Mol Med. 2019 Apr 2;23(6):3878–87. doi: 10.1111/jcmm.14242 (PMC6533474; doi:10.1111/jcmm.14242)

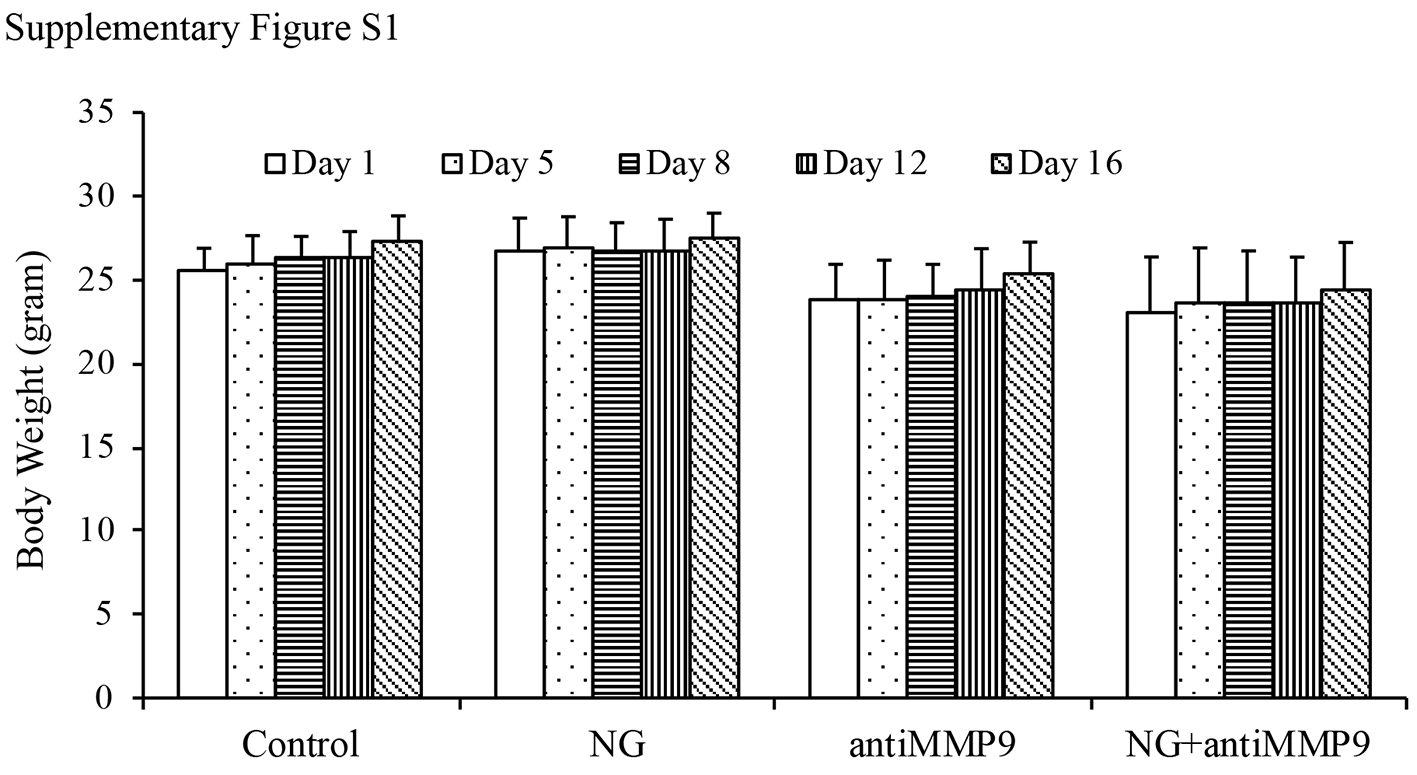

Supplement: Supplementary file 1 [file JCMM-23-3878-s001.tif]

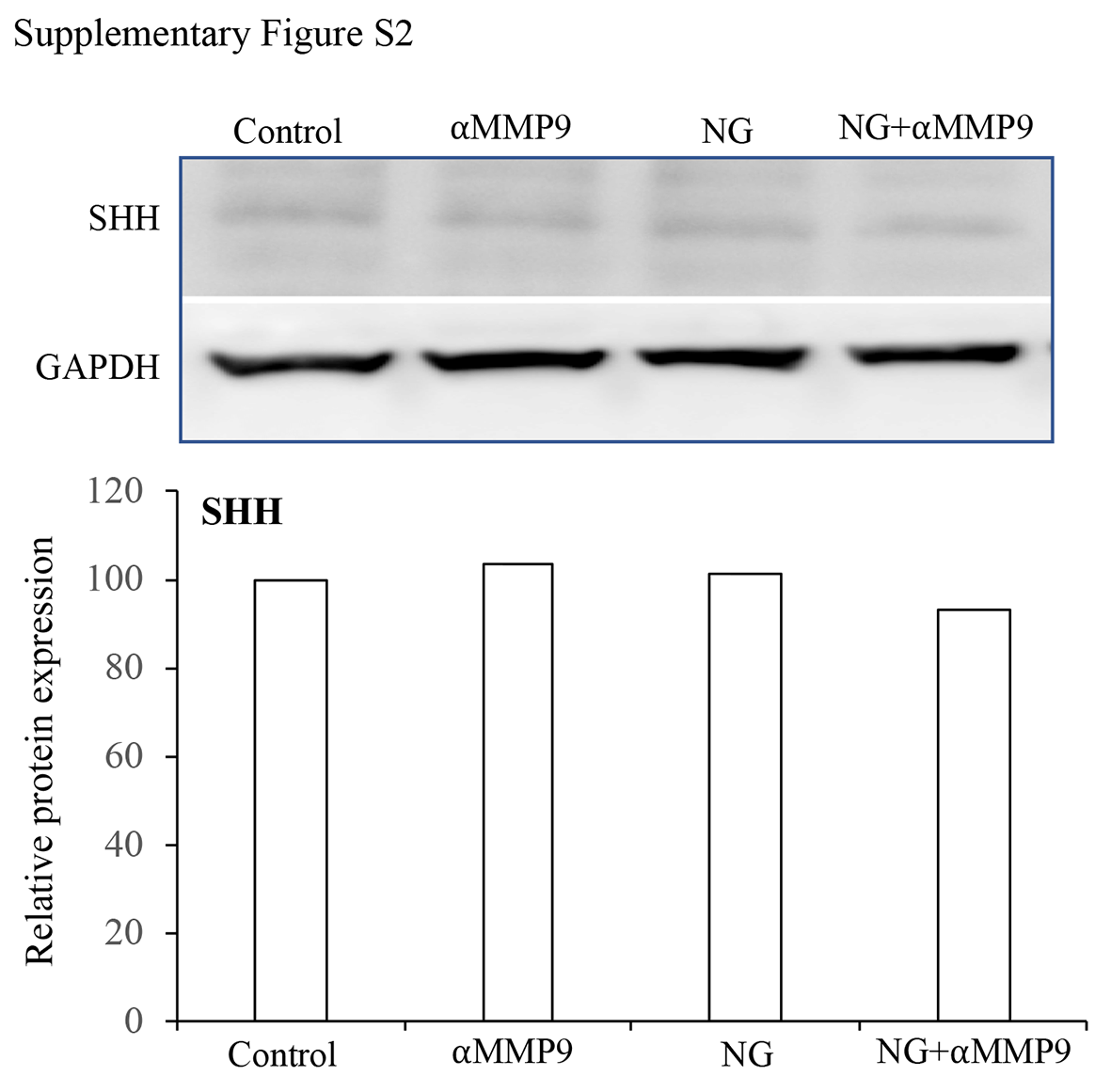

Supplement: Supplementary file 2 [file JCMM-23-3878-s002.tif]
