# Supplementary material for: Therapeutic efficacy of anti‐MMP9 antibody in combination with nab‐paclitaxel‐based chemotherapy in pre‐clinical models of pancreatic cancer
Source: J Cell Mol Med. 2019 Apr 2;23(6):3878–87. doi: 10.1111/jcmm.14242 (PMC6533474; doi:10.1111/jcmm.14242)
Supplement: Supplementary file 3 [file JCMM-23-3878-s003.docx]

**Supplementary Figure Legend:**

**Figure S1**: Mouse body weight in AsPC-1 PDAC cell-derived subcutaneous xenografts. NOD/SCID mice were subcutaneously injected with AsPC-1 cells (7.5 x 10^5^). Two weeks after tumor cell injection, mice were treated intraperitoneally with *nab*-paclitaxel, gemcitabine and anti-MMP9 antibody. Mouse body weight was measured twice a week and presented as a bar chart during the therapy period. Data are representative of the mean values ± standard deviation.

**Figure S2:** Immunoblot analysis of sonic hedgehog (SHH) protein expression after treatment with anti-MMP9 antibody and *nab*-paclitaxel-based chemotherapy: Protein lysates of tumors obtained from intraperitoneal xenografts were separated by SDS-PAGE and the membranes were incubated with antibodies against IL-6 and GAPDH. Protein bands were visualized using the enhanced chemiluminescence reagent. The intensity of bands was quantitated by densitometry and is represented in the bar graph after normalizing values with GAPDH expression.
